# The Brain

**Published:** 1891-06

**Authors:** 


### THE BRAIN.

Remember that the human brain is one of the most delicate, most wonderful, most marvelous structures that the mind can conceive. It is an engine which is only a few inches in diameter, the average weight of which is less than fifty ounces, which contains hundreds of millions of cells and fibers, these cells and fibers varying in thickness from one-three-hundredth to one-millionth of an inch. Every square inch of the gray matter affords substrata for the evolution of at least eight thousand registered and separate ideas; substrata in the whole brain for evolving and registering tens of millions of them, besides the power of recalling them under appropriate stimuli; it transmits thoughts, emotions, sensation and volition, by distinct fibers, whose time working has been ingeniously measured to the fraction of a second. This most wonderful and beautiful piece of mechanism, then, that works so smoothly, so easily, and without friction or pain when in order and not overburdened, needs only to be abused and overtaxed in order to have the nutrition of every part of the body disturbed, and the functions of the various organs rendered morbid. It needs only a certain amount of nervous exhaustion, varying with different people, to open the door to the inroads of neuralgia and its brood of torments, and to many forms of reflex nervous symptoms that render life a burden.—*Ex.*

---

